# The Dark and Light Sides of Empathy: The Clinical Relevance of the Assessment of Cognitive and Affective Empathy Across Negative and Positive Emotions

**DOI:** 10.3390/ejihpe15030038

**Published:** 2025-03-18

**Authors:** Paweł Larionow

**Affiliations:** Faculty of Psychology, Kazimierz Wielki University, 85-064 Bydgoszcz, Poland; pavel@ukw.edu.pl

**Keywords:** affective empathy, anxiety symptoms, cognitive empathy, depression symptoms, empathy, negative emotions, Perth Empathy Scale, positive emotions, psychopathology, well-being

## Abstract

Is empathy a “double-edged sword”? This study aimed to contribute to a more nuanced understanding of the multidimensional empathy construct in the statistical prediction of negative and positive mental health outcomes. More specifically, this research intended to reveal *whether*, *what*, and *how* four individual empathy dimensions (i.e., cognitive empathy for negative emotions, cognitive empathy for positive emotions, affective empathy for negative emotions, and affective empathy for positive emotions) uniquely statistically predicted the levels of anxiety and depression symptoms, as well as well-being. A total of 786 Polish-speaking adults (452 females and 334 males) filled out a series of self-report questionnaires on empathy (the Perth Empathy Scale), anxiety, and depression symptoms, as well as well-being. Adjusting for demographic variables, the frequentist and Bayesian multiple regression analyses revealed that affective empathy dimensions (i.e., abilities to vicariously share others’ emotions) significantly predicted psychopathology symptoms and well-being, whereas cognitive empathy dimensions (i.e., abilities to understand others’ emotions) did not. In particular, higher affective empathy for negative emotions contributed to worse mental health outcomes, whereas higher affective empathy for positive emotions contributed to better mental outcomes. Overall, the results indicated that individual empathy dimensions demonstrated their specific dark and light sides in the statistical prediction of mental illness and well-being indicators, further supporting the clinical relevance of the multidimensional empathy construct.

## 1. Introduction

### 1.1. The Empathy Construct and Its Definition

In the empathy field, there is a variety of definitions of this construct with its numerous subcharacteristics like “empathic concern”, “emotional contagion”, “emotion sharing”, “perspective taking”, etc. (for comprehensive review, see Hall & Schwartz, 2019). In order to avoid confusion as well as to further reconcile theory and measurement (Hall & Schwartz, 2022), this paper uses the below-mentioned commonly used definition.

Empathy is a relatively stable trait, which encompasses the ability to recognise and understand other people’s emotions, as well as to vicariously experience these emotions (Brett et al., 2023). Empathy comprises two main dimensions: *cognitive empathy*, referring to understanding emotions in others; and *affective empathy*, referring to vicariously experiencing other’s emotions (Brett et al., 2023; Thompson et al., 2022; Vallette d’Osia & Meier, 2024). As there are two main groups of emotions, i.e., negative and positive ones, empathy abilities can be assessed across negative and positive emotions (Brett et al., 2023; Morelli et al., 2015). As such, empathy can be characterised by its four interrelated components: (1) cognitive empathy across negative emotions; (2) cognitive empathy across positive emotions; (3) affective empathy across negative emotions; and (4) affective empathy across positive emotions.

In terms of conceptual and operational definitions, the above-mentioned definition of empathy acts as a clear and useful representation of the multidimensional empathy construct. Consequently, this article and its theoretical (i.e., review of research) and empirical parts were based on this understanding of empathy. Such a methodological approach used in this manuscript does not mean discussing different and often inconsistent conceptions and definitions of empathy (Hall & Schwartz, 2022); indeed, it aimed at further reconciling theory and measurements of empathy based on empirically driven backgrounds across different studies, which are following the same above-described definition of empathy.

### 1.2. The Clinical Relevance of Distinguishing Between Cognitive and Affective Empathy

The distinction between cognitive and affective empathy is useful for understanding different psychopathologies (Montag et al., 2020; Ohse et al., 2024), emotion regulation abilities (Thompson et al., 2022), and other psychological phenomena (e.g., personality traits like the Dark Triad; Duradoni et al., 2023). For instance, Thompson et al. (2022) indicated that cognitive and affective empathy were differentially associated with emotion regulation difficulties; that is, higher cognitive empathy was associated with better emotion regulation, whereas higher affective empathy was related to more pronounced emotion regulation difficulties (Thompson et al., 2022). A large body of research, including systematic reviews, has evidenced that people with different psychosomatic pathologies tended to have lower levels of cognitive and/or affective empathy than healthy controls. For instance, such a pattern was observed in patients with anorexia nervosa (Gaggero et al., 2023; Kerr-Gaffney et al., 2019), social anxiety (Pittelkow et al., 2021), and schizophrenia (Bonfils et al., 2016), etc.

As for the associations of cognitive and affective empathy with psychopathological phenomena, several tentative regularities can be noted. For instance, current meta-analytic studies have indicated that affective empathy (but not cognitive empathy) is slightly positively associated with depression psychopathology, revealing the dark side of affective empathy (Yan et al., 2021). The same unfavourable effect of affective empathy was shown in social anxiety, but the favourable one was noted for cognitive empathy, which was slightly negatively associated with clinical social anxiety (Pittelkow et al., 2021). Such unfavourable effects of affective empathy were demonstrated not only among adult samples but also among children. For instance, Bray et al. (2021) examined a sample of 9- and 10-year-old children and demonstrated that affective empathy was associated with higher internalizing symptoms, whereas cognitive empathy was not linked to these symptoms. In contrast, some studies did not indicate links between affective empathy and psychopathology symptoms, whereas negative links between cognitive empathy and these symptoms were pronounced (e.g., Bennik et al., 2019; Zhou & Wu, 2024).

In terms of other correlates, including the ones related to work, higher affective empathy was associated with higher fatigue, but cognitive empathy was not (Vallette d’Osia & Meier, 2024). While higher affective empathy was associated with higher levels of burnout, anxiety, and depression symptoms, higher cognitive empathy was associated with lower of these mental health issues (for review, see Chen et al., 2024). Regarding positive mental health outcomes, cognitive empathy was positively linked to well-being, whereas affective empathy was negatively linked to well-being (Di Girolamo et al., 2017).

In general, the above-mentioned studies indicated that reduced empathy abilities were more prevalent in a range of psychopathologies, with significant positive associations of affective empathy with psychopathology in both adult and children samples. While cognitive empathy seems to be *rather slightly* related to better mental health (e.g., lower levels of symptoms), affective empathy seems to be *rather strongly* related to worse mental health. This evidence highlights the importance of distinguishing the cognitive and affective domains of empathy.

### 1.3. The Role of Emotional Valence: The Distinction Between Cognitive and Affective Empathy Across Negative and Positive Emotions

Recent evidence suggests that more specific and robust conclusions can be made if the valence of emotions (i.e., negative vs. positive) is taken into account when studying the link between empathy and other psychological phenomena (Morelli et al., 2015). For instance, current clinical studies revealed that deficits in cognitive empathy and affective empathy for negative and positive emotions were associated with specific personality pathologies (Ohse et al., 2024). Brett et al. (2024) have recently noted that self-report autistic traits were related to higher affective empathy for negative emotions and lower affective empathy for positive emotions, supporting the valence-specific empathy imbalance hypothesis of autism. This overview shows that assessing both cognitive and affective empathy across negative and positive emotions separately is highly relevant to understanding of psychopathology.

### 1.4. The Perth Empathy Scale: A New Multidimensional Empathy Measure and Its Clinical Utility

Due to a variety of definitions of empathy and their corresponding measures (Hall & Schwartz, 2019, 2022), it is essential to elaborate on the ones upon which this study was based. This is the Perth Empathy Scale (PES), a psychometric tool for assessing the multidimensional empathy construct across affective (i.e., vicariously experiencing other’s emotions) and cognitive (i.e., understanding emotions in others) domains, as well as negative and positive emotions (Brett et al., 2023). When assessing affective (e.g., “When I see or hear someone who is happy, it makes me feel happy too”) and cognitive (e.g., “Just by seeing or hearing someone, I know if they are feeling happy”) empathy domains, the PES uses five positive and five negative emotions, making the assessment balanced and comprehensive (Brett et al., 2023).

Originally developed in English, the PES has been introduced in 2022 (Brett et al., 2023). To date, there are only several cross-cultural validation studies, including Polish (Larionow & Preece, 2023) and Chinese studies (Ye et al., 2024), as well as clinical research (e.g., Brett et al., 2024). Specifically, in the Chinese validation study (Ye et al., 2024), patients with major depressive disorder showed significantly lower scores of each PES subscale than healthy controls. As for the predictive role of the PES scores in psychopathology symptoms, affective empathy dimensions across negative and positive emotions were significant predictors of anxiety and depression symptoms (Larionow & Preece, 2023). In other words, people with higher abilities to vicariously experience other’s negative emotions tended to have higher levels of anxiety and depression symptoms, and the ones with higher abilities to vicariously experience other’s positive emotions tended to have lower levels of these symptoms (Larionow & Preece, 2023).

Overall, these results consistently indicate good clinical utility of the PES across non-clinical and clinical samples and suggest that the PES is a promising measure of the multidimensional empathy construct. As such, this scale was considered a useful choice in the current study to further explore the specific patterns of associations between empathy and mental health correlates (e.g., Larionow & Preece, 2023).

### 1.5. The Current Study

To increase our knowledge about the role of the individual empathy dimensions not only in psychopathology but also in maintaining well-being, this study aimed to reveal the specific associations between four empathy dimensions and a series of illness and well-being indicators simultaneously. This is aligned with the dual-factor models of mental health (Magalhães, 2024), as well as ideas about common and specific predictors of positive (i.e., well-being) and negative (i.e., illness) mental health indicators (Karademas, 2007).

The above-described studies suggest the clinical relevance of the PES, chiefly its affective empathy dimensions for predicting illness. However, no studies have yet investigated the role of different empathy dimensions in predicting well-being. Thus, in order to better understand how empathy abilities can contribute to mental health, this study was devoted to a question on *whether*, *which*, and *how* individual empathy dimensions are uniquely linked to illness and well-being.

Therefore, the aim of this study was to explore the clinical relevance of assessment of cognitive and affective empathy across negative and positive emotions via examining the predictive ability of these empathy dimensions for anxiety and depression symptoms, as well well-being. To investigate this, both frequentist and Bayesian multiple linear regression analyses, with sociodemographic variables used as controlling variables, were applied. Based on past studies (Larionow & Preece, 2023), the two following and interrelated hypotheses (H) were put forward:

**H1.**  *Affective empathy dimensions scores would be significant statistical predictors of mental health outcomes, with affective empathy across negative emotions associated with worse outcomes and affective empathy across positive emotions associated with better outcomes in multiple regression analysis*.

**H2.**  *Compared to affective empathy dimensions, cognitive empathy dimensions across negative and positive emotions would be similarly but less pertinently associated with mental health outcomes (or even would not demonstrate significant associations) in multiple regression analysis*.

## 2. Materials and Methods

### 2.1. Procedure

This study was conducted in accordance with the Declaration of Helsinki Ethical Principles. The Ethics Committee of the Faculty of Psychology of Kazimierz Wielki University approved the study (No. 1/13.06.2022). To ensure research transparency, it should be noted that these data are a part of a large project on empathy, alexithymia, and mental health outcomes. A small part of these data (i.e., only PES scores) has been published in Larionow et al.’s (2024) paper. This large project was devoted to different research questions, with no overlapping analyses. Each research question was supposed to be comprehensively addressed in a separate publication (Supak Smolcić, 2013).

From October 2023 to February 2024, Polish-speaking people aged 18 years or over were invited to complete a study via Facebook and Instagram. In these social networking sites, a link with an invitation to complete this online anonymous and voluntary survey, which was hosted on the Google Forms platform, was posted. The survey included an informed consent form as well as a demographic form with a set of short psychological questionnaires. Written informed consent was provided by participants digitally before completing the survey. Without antecedent signing of written informed consent, no access to the questionnaires was provided. All responses on the questionnaires were obligatory; thus, there were no missing data.

The inclusion criteria were Polish-speaking people aged 18 years or over, who signed their informed consent for study participation. These were people from a general Polish population, without any specific characteristics that were targeted during recruitment. The exclusion criteria were people who identified their gender outside of female and male identities. In contemporary research practices, the visibility of gender identity in studies should be recognised (Larionow & Mazur, 2024). However, in this study, people with non-binary gender categories were excluded for several reasons. First, in this study, potential gender effects on the variables were accounted for; therefore, for interpretability reasons, only data on females and males were used in statistical analysis. Second, the number of people with non-binary gender categories was low (*n* = 8), which made it impossible to process these limited data in a statistical analysis. As such, a total of 794 participants replied to the consent form, and the data of 8 individuals (i.e., 6 non-binary people, 1 transgender male, and 1 person with unidentified gender) were considered invalid based on the exclusion criteria. After the exclusion of these 8 people, the final sample comprised 786 participants.

### 2.2. Participants

The sample was 786 Polish-speaking adults (452 females and 334 males) recruited from the general population in Poland, with ages ranging from 18 to 76 years (*M* = 28.63; *SD* = 12.58). Detailed demographic characteristics of the participants are displayed in Table 1.

### 2.3. Measures

#### 2.3.1. The Sociodemographic Form

All participants filled out a sociodemographic questionnaire on age, gender, education degree, and relationship status.

#### 2.3.2. The Perth Empathy Scale (PES)

The PES is a 20-item self-report measure of empathy (Brett et al., 2023). The PES has four subscales: Negative Cognitive Empathy (e.g., “Just by seeing or hearing someone, I know if they are feeling sad”), Positive Cognitive Empathy (e.g., “Just by seeing or hearing someone, I know if they are feeling happy”), Negative Affective Empathy (e.g., “When I see or hear someone who is sad, it makes me feel sad too”), and Positive Affective Empathy (e.g., “When I see or hear someone who is happy, it makes me feel happy too”). There are also three composite subscale scores: General Cognitive Empathy, General Affective Empathy, and General Empathy (Total Score). All PES items are scored on a 5-point Likert scale, ranging from 1 (“almost never”) to 5 (“almost always”). All PES subscale and composite scores are calculated by summing appropriate items. Higher scores indicate higher levels of empathy abilities.

For this study, the Polish version of the PES, which has previously shown good psychometric properties in a Polish general community sample (Larionow & Preece, 2023), was used. The questionnaire has previously demonstrated good factorial and concurrent validity, as well as internal consistency (i.e., McDonald’s omega and Cronbach’s alpha coefficients of ≥0.80) and test–retest reliability. The PES scores were statistically separable from psychological distress, supporting good discriminant validity (Larionow & Preece, 2023). In the current study, the omega and alpha reliability coefficients for PES subscale and composite scores were ≥0.78 (for details, see Table 2), suggesting acceptable internal consistency reliability.

#### 2.3.3. The Patient Health Questionnaire-4 (PHQ-4)

The PHQ-4 is a 4-item self-report measure of anxiety and depression symptoms, which are being assessed over the previous two weeks (Kroenke et al., 2009). The PHQ-4 has two subscales: anxiety (e.g., “Feeling nervous, anxious, or on edge”) and depression (e.g., “Feeling down, depressed, or hopeless”). There is also a total PHQ-4 score, which represents an overall level of the psychopathology symptoms. All PHQ-4 items are scored on a 4-point Likert scale, ranging from 0 (“not at all”) to 3 (“nearly every day”). The PHQ-4 subscale scores, as well as the total score, are calculated by summing appropriate items. Higher scores indicate higher levels of measured symptoms.

For this study, the Polish version of the PHQ-4 (Larionow & Mudło-Głagolska, 2023), which has previously shown good psychometric properties in a Polish general community sample, was used. In the Polish validation study of the PHQ-4, internal consistency reliability was acceptable, with McDonald’s omega coefficients of ≥0.73 in samples of Polish females and males (Larionow & Mudło-Głagolska, 2023). In the current study, omega and alpha reliability coefficients for PHQ-4 subscale scores and the total score were ≥0.77 (for details, see Table 2), suggesting acceptable internal consistency reliability.

#### 2.3.4. The WHO-Five Well-Being Index (WHO-5)

The WHO-5 is a 5-item self-report measure of well-being, which is being assessed over the previous two weeks (Topp et al., 2015; World Health Organization. Regional Office for Europe, 1998). The WHO-5 has a total score, which represents an overall level of positive well-being. All WHO-5 items (e.g., “I feel cheerful and in good spirits”) are scored on a 6-point Likert scale, ranging from 0 (“at no time”) to 5 (“all the time”). The WHO-5 score is calculated by summing all the items. A higher score indicates a higher level of well-being.

For this study, the Polish version of the WHO-5, which has previously shown good psychometric properties in a Polish general community sample (Larionow, 2023) and in a clinical one (Cichoń et al., 2020), was used. The Polish WHO-5 has demonstrated alpha and omega reliability coefficients of 0.85 in a general community sample of Poles (Larionow, 2023). Similarly, in the current study, these coefficients were 0.86 (for details, see Table 2), suggesting good internal consistency reliability.

### 2.4. Analytic Strategy

Statistical analyses were carried out using *Statistica* v. 13.3 and *JASP* v. 0.19.0.0. Descriptive statistics and Pearson correlations between the study variables were computed. For assessing internal consistency reliability, a Bayesian reliability analysis was conducted, and McDonald’s omega and Cronbach’s alpha coefficients with 95% credible intervals were computed (Pfadt et al., 2023).

In order to reveal whether and which empathy dimensions are significant predictors of psychopathology and well-being, a multiple regression analysis, controlling for sociodemographic variables (for review, see Ross & Willson, 2017), was conducted. For this analysis, both frequentist and Bayesian approaches were applied. It should be noted that the word “predictor” used in this paper acts as a statistical term; therefore, this word does not suggest causality.

Across all regression analyses, anxiety and depression symptoms, as well as well-being, were dependent variables, whereas sociodemographic variables (i.e., sex, age, education, and relationship status) and four PES subscale scores were predictors. In the frequentist multiple regression analysis, a hierarchical regression with two steps was used. In the first step, sociodemographic variables were added as predictors. In the second step, four empathy dimensions were added as predictors.

A Bayesian analysis with its interpretation was conducted following common guidelines (van Doorn et al., 2021). More specifically, in the Bayesian multiple regression analysis (Bergh et al., 2021), all sociodemographic variables and four PES subscale scores were added as predictors. The ten best models with the highest posterior probability were presented. In order to evaluate the relevance of individual predictors, the model-averaged posterior summary for linear regression coefficients were shown. To reveal the most pertinent predictors, BF_inclusion_ parameters were analysed. Higher BF_inclusion_ values above 1 suggest increasing the importance of the variable as a relevant predictor in the regression model. Values > 100 indicate extreme evidence for inclusion the predictor into the model. In contrast, BF_inclusion_ values around 1 indicate no evidence on the relevance of the predictor in the regression model. Lower BF_inclusion_ values below 1 suggest decreasing the importance of the variable as a relevant predictor in the model. Values < 0.01 suggest extreme evidence for excluding the predictor from the regression model. A more nuanced categorisation of the strength of a Bayes factor is posted in Bergh et al.’s (2021) paper.

## 3. Results

Table 2 demonstrates descriptive statistics and reliability coefficients for the study variables.

Internal consistency reliability was good for all questionnaire scores, with McDonald’s Omega and Cronbach’s Alpha coefficients of 0.77 and above (see Table 2). Pearson correlations between the study variables are displayed in Supplementary Table S1. Bivariate correlation analysis indicated that Negative Cognitive Empathy was slightly positively associated with anxiety symptoms (*r* = 0.10, *p* < 0.01) and well-being (*r* = 0.11, *p* < 0.01). Positive Cognitive Empathy was negatively associated with depression symptoms (*r* = −0.10, *p* < 0.01) and positively with well-being (*r* = 0.22, *p* < 0.001). Negative Affective Empathy was positively associated with anxiety symptoms (*r* = 0.28, *p* < 0.001) and depression symptoms (*r* = 0.14, *p* < 0.001); however, it was not associated with well-being (*r* = −0.03, *p* > 0.05). Positive Affective Empathy was negatively associated with depression symptoms (*r* = −0.19, *p* < 0.001) and positively with well-being (*r* = 0.29, *p* < 0.001; for details, see Supplementary Table S1).

Controlling for sociodemographic variables, the frequentist linear regression analyses indicated that Negative Affective Empathy and Positive Affective Empathy scores were significant predictors of anxiety and depression symptoms, as well as well-being, whereas Negative Cognitive Empathy and Positive Cognitive Empathy scores were not significant predictors (see Table 3).

Higher levels of Negative Affective Empathy were associated with higher frequency of anxiety and depression symptoms, as well as lower levels of well-being. In contrast, higher levels of Positive Affective Empathy were associated with lower frequency of anxiety and depression symptoms, as well as higher levels of well-being. Beyond sociodemographic variables, empathy dimensions explained 10.42% of variance of anxiety symptoms, 9.67% of variance of depression symptoms, as well as 13.19% of variance of well-being (see Table 3).

The Bayesian regression analyses supported the linear regression analysis results. Overall, almost the same set of relevant predictors was shown. There were four relevant predictors of anxiety symptoms, namely, sex, age, Negative Affective Empathy, and Positive Affective Empathy (see Table 4 and Supplementary Table S2), with extreme evidence of the relevance of these two empathy dimensions (BF_inclusion_ > 100 in both cases).

There were three relevant predictors of depression symptoms, namely, age, Negative Affective Empathy, and Positive Affective Empathy (see Table 5 and Supplementary Table S3), with extreme evidence of the relevance of these two empathy dimensions (BF_inclusion_ > 100 in both cases).

In contrast, there were five relevant predictors of well-being, namely, sex, age, Positive Cognitive Empathy, Negative Affective Empathy, and Positive Affective Empathy (see Table 6 and Supplementary Table S4), with extreme evidence of the relevance of Negative Affective Empathy and Positive Affective Empathy (BF_inclusion_ > 100 in both cases) and anecdotal evidence of the relevance of Positive Cognitive Empathy (BF_inclusion_ = 2.84).

Taking into account all the frequentist and Bayesian regression analyses, Negative Affective Empathy and Positive Affective Empathy were the strongest unique predictors of psychopathology symptoms and well-being, whereas two cognitive empathy dimensions were irrelevant predictors.

## 4. Discussion

The aim of this study was to reveal *whether*, *which*, and *how* individual empathy traits were uniquely associated with illness and well-being. Overall, the clinical relevance of the empathy construct was consequently supported in a series of multiple regression analyses conducted using the frequentist and Bayesian approaches. As such, following Tone and Tully’s (2014) ideas about empathy as a “risky strength”, this analysis has elaborated *whether*, *which*, and *how* different empathy components were associated with one’s mental states.

### 4.1. Negative and Positive Affective Empathy as Strong Predictors of Illness and Well-Being

Overall, the bivariate correlation analysis demonstrated that four empathy dimensions were statistically significantly associated with psychopathology symptoms and well-being, albeit with no statistically significant link between affective empathy for negative emotions and well-being. Therefore, the empathy construct, as measured with the PES, showed theoretically reasonable associations with mental health indicators, supporting previous findings (Larionow & Preece, 2023).

The frequentist multiple regression analysis, controlling for demographic variables, has demonstrated that affective empathy dimensions were significant predictors of psychopathology symptoms and well-being, whereas cognitive empathy dimensions were not significant predictors. In other words, understanding other’s emotions is not related to illness and well-being indicators of mental health, whereas vicariously sharing others’ emotions can contribute to mental health. The more people vicariously experience others’ negative emotions, the more often they experience anxiety and depression symptoms. In contrast, the more people vicariously experience other’s positive emotions, the more often they experience well-being. The same conclusions can be derived from the results of the Bayesian multiple regression analysis, thus further supporting the clinical relevance of affective empathy across negative and positive emotions for predicting not only illness indicators (Brett et al., 2024; Larionow & Preece, 2023), but also positive ones. A lack of statistical associations between cognitive empathy dimensions and affective outcomes in the regression analysis could be explained by the fact that understanding other’s emotions (a cognitive process) does not necessarily trigger a specific emotional reaction.

Considering potential mechanisms linking affective empathy with psychopathology, from the perspective of the vulnerability–stress models, frequent exposures to negative emotions can lead to the development of psychopathology, in particularly, when predisposing factors exist (Chaplin & Cole, 2005; Ingram & Luxton, 2005). As affective empathy reflects a process of sharing and experiencing others’ both negative and positive emotions, frequent exposures to such emotions can lead to both negative and positive mental health outcomes, respectively. Among potential vulnerability factors, alexithymia (i.e., difficulties in assessing one’s own mental states) and emotion regulation seem to play a crucial role in the explaining the link between empathy and psychopathology as problematic (both low and high) levels of empathy seem to be related to alexithymia and emotion dysregulation (Schipper & Petermann, 2013). Alexithymia is considered a transdiagnostic risk factor for a wide range of psychopathologies and dysfunctional interpersonal relationships (Luminet & Nielson, 2025). Emotion dysregulation is treated as a precursor of psychopathology (Schipper & Petermann, 2013) and, at that, alexithymia and emotion regulation are strongly associated (Mehta et al., 2025; Preece et al., 2023). People with high levels of affective empathy for negative emotions and low levels of affective empathy for positive emotions might experience worse mental health due to difficulties in processing their own emotions (i.e., high alexithymia), which, in turn, impairs emotion regulation abilities across negative emotions (i.e., downregulation of their own negative affects caused by others’ negative emotional states) and positive emotions (i.e., upregulation of their own positive affects caused by others’ positive emotional states; Brett et al., 2023). That is, alexithymia and emotion regulation seem to act as moderators in the relationship between empathy and mental health outcomes. For instance, Powell (2018) proved this idea when demonstrating that people with higher levels of affective empathy, who effectively managed their emotions, did not experience elevated levels of psychological distress. Hence, including alexithymia and emotion regulation in research on empathy is of great potential.

One of the most interesting results in this research was that affective empathy for positive emotions was a *stronger* negative predictor of depression symptoms than affective empathy for negative emotions being a positive predictor of depression symptoms. These results highlight the specific relevance of reduced empathy abilities in predicting depression symptoms and imply that reduced ability to share others’ *positive* emotions might be treated as a *stronger* risk factor for depression than elevated ability to share others’ *negative* emotions. These results support current literature reviews about depression and its difficulties in the regulation of positive emotions (i.e., people with depression habitually attempt to downregulate and infrequently attempt to upregulate their positive emotions; Vanderlind et al., 2020). It has been shown that people with recurrent depression had reduced emodiversity than healthy controls, suggesting that experiencing a more diverse range of positive emotions could be helpful in depression (Werner-Seidler et al., 2020). Therefore, the development of empathy abilities across positive emotions might be considered relevant for potential prevention of depression symptoms.

Overall, the contemporary evidence on the role of cognitive and affective empathy across negative and positive emotions in mental health is limited. However, the results of the current study are promising as they revealed that different problematic levels of individual empathy domains were associated with more or less pertinently with corresponding mental health outcomes. Hence, from this perspective, examining psychological mechanisms linking different empathy dimensions with illness and well-being is of great interest.

### 4.2. The Practical and Theoretical Value of the Study

Based on the current results and above-described studies, it is crucial to analyse potential practical implications of this research. Overall, this study has shown that affective empathy for negative emotions and affective empathy for positive emotions were important predictors of both illness and well-being, whereas cognitive empathy (across both negative and positive emotions) was not. This indicates that psychological interventions should principally focus on the harmonious development of abilities to vicariously experience others’ emotions. Based on these data, it can be concluded that the most pertinent aim in these interventions is to develop the ability to vicariously experience others’ *positive* emotions, as they were associated with less mental health problems and higher well-being. Through the mechanism of susceptibility to positive emotional contagion (Herrando & Constantinides, 2021; Marx et al., 2024), these positive emotions could be expanded to other people, providing overall positive cumulative effects for all parties of interpersonal relationships (Zaki, 2020).

As for the development of abilities to vicariously experience others’ *negative* emotions, precautions should be taken into account as these abilities were associated with more mental health problems and lower well-being. Despite the fact that empathy is basically considered a positive prosocial personality trait (Depow et al., 2021), such dark sides of affective empathy for negative emotions should be taken into account in psychological assessment. The reasons and potential negative consequences of higher levels of affective empathy for negative emotions should be analysed with a patient (or a client). In the first place, it should be examined whether higher levels of affective empathy for negative emotions cause intra- and interpersonal problems in a patient. If sharing others’ negative emotions leads to worse emotional functioning, a deep understanding of this state (i.e., through assessment of alexithymia, emotional reactivity, emotion regulation difficulties, etc.) and prevention measures should be applied. This is important not only for a patient but also for people from the patient’s personal environment, who might become infected with these negative emotions (Zaki, 2020).

The current study has further demonstrated good clinical utility of the PES (Brett et al., 2024; Larionow & Preece, 2023; Ye et al., 2024) and directly connects it to clinical practice, showing its usefulness in psychological assessment (Larionow & Preece, 2023). As there are Polish norms for the PES (Larionow et al., 2024), it facilitates the use of this scale in research and practice. The PES can be integrated into different mental health programs. For instance, developing empathetic skills is useful in health and social care professionals (Moudatsou et al., 2020); therefore, the current results about the nuanced potential effects of individual empathy traits for mental health could provide practitioners with more robust ideas of how to develop empathy in health and social workers. The results might be also adapted for other populations in different settings (e.g., students/teachers in the educational context; Aldrup et al., 2022), providing the long-term applicability for public health.

The clinical relevance of the dual-factor models of mental health, with negative (i.e., illness or symptoms of psychopathology) and positive (i.e., well-being) mental health indicators (Magalhães, 2024) was supported. According to these models, the current study has demonstrated that it is essential to examine the role of different empathy traits in predicting illness and well-being concomitantly, revealing common and specific predictors of these negative and positive mental health indicators among different empathy dimensions (Karademas, 2007).

### 4.3. Strengths and Limitations of the Study

This study represents a good contribution to the field of empathy and in general clinical and personality psychology, and individual differences disciplines. The main strengths of the study are (1) a large sample from a general community, with almost the same gender ratio; (2) the use of two comprehensive statistical approaches (i.e., frequentist and Bayesian multiple regression analysis), with controlling sociodemographic variables; and (3) the application of the dual-factor models of mental health in examining the clinical relevance of the multidimensional empathy construct.

However, this study has several limitations. First, this research was based on a cross-sectional study design, which does not allow us to examine the directionality of the analysed relationships. Based on the theory and empirical evidence that the PES measures empathy as relatively stable trait (Larionow & Preece, 2023; Ye et al., 2024), this limitation regarding the cross-sectional study design was mitigated. Therefore, empathy served as the predictor variable, whereas psychopathology symptoms and well-being were the dependent variables (the terms “predictor” and “dependent variable” serve as statistical terms in this paper).

Second, this was a self-report study, with the recruitment of participants characterised by self-selection bias. To mitigate this limitation, diversifying social networking sites during the recruitment process was used.

Finally, by its nature, self-report measures capture people’s self-efficacy or beliefs about their abilities and dispositions. So, whilst this paper uses the term “ability” because empathy is often regarded as an ability (Decety et al., 2016; Hall & Schwartz, 2019; Leiberg & Anders, 2006), a performance measure of empathy was not used in this study, and the current results should be interpreted in that light.

### 4.4. Future Directions

This was a cross-sectional study; therefore, future studies should use a longitudinal study design to further examine the cause-and-effect associations between empathy and other correlates. Including other variables of interest (e.g., burnout) would be also beneficial.

The examined sample was not clinical. As such, including various clinical samples is required to examine the universality of the studied relationships between empathy and mental health outcomes. Also, examining the role of different empathy dimensions in specific occupational groups (e.g., doctors, teachers) is important to provide more specific recommendations of how to develop and manage empathy skills effectively.

This research was based on a variable-centred approach. Therefore, in future studies, the use of person-centred approaches (e.g., latent profile analysis), allowing us to reveal subgroups of people with the same characteristic patterns of empathy (i.e., specific empathy profiles), would be beneficial in examining whether the specific configurations of empathy dimensions exist and how they are related to different mental health outcomes. Such an approach has shown its high effectiveness in examining emotion-related variables (e.g., Larionow et al., 2025); therefore, it might be promisingly applied to the empathy field. As problematic levels of individual empathy domains can be both elevated and lowered (Schipper & Petermann, 2013) and can potentially co-exist in the same individual, examining profiles of empathy using person-centred approaches is required in future research to reveal whether the patterns observed in this variable-centred study would be replicated in a person-centred study.

## 5. Conclusions

This study has contributed to a more nuanced understanding of *whether*, *which*, and *how* different empathy traits are related to anxiety and depression symptoms, as well as well-being. The frequentist and Bayesian multiple regression analyses consequently supported the idea that affective empathy for negative emotions and affective empathy for positive emotions were significant predictors of mental health outcomes, whereas cognitive empathy for negative emotions and affective empathy for positive emotions were not. As such, different empathy domains relate differentially to mental health indicators. Apparently, empathy is a psychological construct of a “double-edged sword” nature, which can be clearly understood when examining its individual domains.

These results highlight the clinical significance of sharing others’ negative and positive emotions in predicting mental health. In other words, people with high capability in sharing others’ negative emotions might be at higher risk for mental health issues and low well-being, whereas people with high capability in sharing others’ positive emotions might be at lower risk for mental health issues and could experience higher well-being.

Moreover, this study has further indicated the clinical utility of the PES (Brett et al., 2024; Larionow et al., 2024; Larionow & Preece, 2023; Ye et al., 2024) and its potential applicability in different clinical and non-clinical contexts. This study has also demonstrated that examining the multidimensional nature of the empathy construct, with its different traits having dark and light sides with respect to negative and positive mental health, reinforces the theoretical and practical importance of studying emotional valence in emotion-based psychological constructs and using dual-factor models of mental health.

## Figures and Tables

**Table 1:** Demographic characteristics of the participants.

Variables	Demographic Categories	*n*	%
Age	*M* = 28.63, *SD* = 12.58, median = 23.00, min. = 18, max. = 76	786	100
Gender	Females	452	57.51
	Males	334	42.49
Education	Primary	52	6.62
	Vocational	48	6.11
	Secondary	438	55.73
	Higher	248	31.55
Relationship status	Single	376	47.84
	In a relationship	410	52.16

**Table 2:** Descriptive statistics and Bayesian internal consistency reliability coefficients for the study variables (*n* = 786).

Variables	McDonald’s Omega with 95% Credible Interval	Cronbach’s Alpha with 95% Credible Interval	*M*	*SD*	Skewness	Kurtosis	Minimum	Maximum
PES Negative Cognitive Empathy	0.88 (0.86; 0.89)	0.88 (0.86; 0.89)	19	4.36	−0.68	0.36	5	25
PES Positive Cognitive Empathy	0.86 (0.84; 0.87)	0.86 (0.84; 0.87)	19.18	4.19	−0.66	0.36	5	25
PES Negative Affective Empathy	0.78 (0.75; 0.80)	0.78 (0.75; 0.80)	12.5	4.13	0.3	−0.2	5	25
PES Positive Affective Empathy	0.82 (0.80; 0.84)	0.82 (0.80; 0.84)	14.62	4.46	−0.06	−0.29	5	25
PES General Cognitive Empathy	0.92 (0.92; 0.93)	0.92 (0.92; 0.93)	38.18	8.16	−0.68	0.49	10	50
PES General Affective Empathy	0.85 (0.83; 0.86)	0.85 (0.83; 0.86)	27.11	7.47	0.02	0.14	10	50
PES General Empathy (Total Score)	0.89 (0.88; 0.90)	0.90 (0.89; 0.91)	65.3	13.09	−0.47	0.75	20	100
PHQ-4 Anxiety	0.77 (0.74; 0.80)	0.77 (0.73; 0.80)	3.52	1.9	−0.1	−1.26	0	6
PHQ-4 Depression	0.81 (0.79; 0.84)	0.81 (0.79; 0.84)	3.41	2.02	−0.1	−1.29	0	6
PHQ-4 Total Score	0.86 (0.84; 0.87)	0.86 (0.84; 0.87)	6.93	3.61	−0.1	−1.2	0	12
WHO-5 Well-Being	0.86 (0.84; 0.88)	0.86 (0.85; 0.88)	8	4.94	0.77	0.3	0	25

**Table 3:** Regression models for predicting anxiety and depression symptoms, as well as well-being (*n* = 786).

Models	Predictors	Anxiety Symptoms Prediction (PHQ-4 Anxiety Scores as the Dependent Variable)			Depression Symptoms Prediction (PHQ-4 Depression Scores as the Dependent Variable)			Well-Being Prediction (WHO-5 Scores as the Dependent Variable)		
		Beta	*t*	*p*	Beta	*t*	*p*	Beta	*t*	*p*
Step 1	Sex	**−0.13**	**−3.88**	**<0.001**	−0.02	−0.48	0.629	**0.08**	**2.36**	**0.019**
	Age	**−0.25**	**−6.36**	**<0.001**	**−0.21**	**−5.44**	**<0.001**	**0.15**	**3.75**	**<0.001**
	Education	0.01	0.26	0.791	0	−0.11	0.914	0.04	1	0.317
	Relationship status	0.03	0.8	0.424	−0.06	−1.57	0.117	−0.01	−0.22	0.822
Step 2	Sex	**−0.09**	**−2.75**	**0.006**	−0.001	−0.15	0.879	**0.1**	**2.9**	**0.004**
	Age	**−0.21**	**−5.76**	**<0.001**	**−0.19**	**−5.01**	**<0.001**	**0.13**	**3.56**	**<0.001**
	Education	−0.03	−0.82	0.415	−0.03	−0.77	0.442	0.04	1.23	0.219
	Relationship status	0.03	0.9	0.367	−0.05	−1.44	0.149	−0.02	−0.73	0.465
	PES Negative Cognitive Empathy	0.08	1.34	0.179	0.01	0.17	0.862	−0.02	−0.29	0.774
	PES Positive Cognitive Empathy	−0.04	−0.66	0.512	0	0.01	0.993	0.11	1.59	0.112
	PES Negative Affective Empathy	**0.36**	**9.06**	**<0.001**	**0.3**	**7.42**	**<0.001**	**−0.22**	**−5.35**	**<0.001**
	PES Positive Affective Empathy	**−0.24**	**−5.52**	**<0.001**	**−0.34**	**−7.56**	**<0.001**	**0.37**	**8.3**	**<0.001**
	Model parameters for Step 1	*F*(4,781) = 16.78, *p* < 0.001, adj. R^2^ = 7.44%			*F*(4,781) = 11.50, *p* < 0.001, adj. R^2^ = 5.08%			*F*(4,781) = 7.38, *p* < 0.001, adj. R^2^ = 3.15%		
	Model parameters for Step 2	*F*(8,777) = 22.33, *p* < 0.001, adj. R^2^ = 17.86%			*F*(8,777) = 17.98, *p* < 0.001, adj. R^2^ = 14.75%			*F*(8,777) = 20.16, *p* < 0.001, adj. R^2^ = 16.34%		
	Δ adj. R^2^ between Steps 2 and 1	10.42%			9.67%			13.19%		

**Table 4:** Model-averaged posterior summaries of coefficients for predicting anxiety symptoms (*n* = 786).

Coefficient	P(incl)	P(excl)	P(incl|data)	P(excl|data)	BF_inclusion_	*M*	*SD*	95% Credible Interval	
								Lower	Upper
Intercept	1	0	1	0	1	3.52	0.06	3.4	3.63
Sex	0.5	0.5	0.94	0.06	15.46	−0.35	0.15	−0.59	0
Age	0.5	0.5	1	9.41 × 10^−8^	1.06 × 10^+7^	−0.03	5.13 × 10^−3^	−0.04	−0.02
Education	0.5	0.5	0.29	0.71	0.41	−0.02	0.05	−0.17	0.05
Relationship status	0.5	0.5	0.31	0.69	0.45	0.04	0.09	−0.05	0.32
Negative Cognitive Empathy	0.5	0.5	0.41	0.59	0.7	0.01	0.02	−72.6	0.06
Positive Cognitive Empathy	0.5	0.5	0.29	0.71	0.41	−14.9	0.02	−0.05	0.03
Negative Affective Empathy	0.5	0.5	1	5.55 × 10^−16^	2.73 × 10^+16^	0.17	0.02	0.13	0.2
Positive Affective Empathy	0.5	0.5	1	1.86 × 10^−7^	5.38 × 10^+6^	−0.1	0.02	−0.14	−0.08

**Table 5:** Model-averaged posterior summaries of coefficients for predicting depression symptoms (*n* = 786).

Coefficient	P(incl)	P(excl)	P(incl|data)	P(excl|data)	BF_inclusion_	*M*	*SD*	95% Credible Interval	
								Lower	Upper
Intercept	1	0	1	0	1	3.41	0.07	3.27	3.52
Sex	0.5	0.5	0.14	0.86	0.17	−19.6	0.05	−0.15	0.1
Age	0.5	0.5	1	1.69 × 10^−6^	593,161.38	−0.03	5.54 × 10^−3^	−0.04	−0.02
Education	0.5	0.5	0.18	0.82	0.21	−0.01	0.05	−0.13	0.06
Relationship status	0.5	0.5	0.28	0.72	0.4	−0.06	0.11	−0.36	4.72 × 10^−3^
Negative Cognitive Empathy	0.5	0.5	0.15	0.85	0.17	6.39 × 10^−4^	7.80 × 10^−3^	−0.01	0.02
Positive Cognitive Empathy	0.5	0.5	0.14	0.86	0.17	3.51 × 10^−4^	8.57 × 10^−3^	−0.02	0.01
Negative Affective Empathy	0.5	0.5	1	3.68 × 10^−12^	2.72 × 10^+11^	0.14	0.02	0.11	0.18
Positive Affective Empathy	0.5	0.5	1	2.91 × 10^−13^	3.43 × 10^+12^	−0.15	0.02	−0.19	−0.12

**Table 6:** Model-averaged posterior summaries of coefficients for predicting well-being (*n* = 786).

Coefficient	P(incl)	P(excl)	P(incl|data)	P(excl|data)	BF_inclusion_	*M*	*SD*	95% Credible Interval	
								Lower	Upper
Intercept	1	0	1	0	1	8	0.16	7.66	8.27
Sex	0.5	0.5	0.96	0.04	26.14	0.96	0.38	0	1.5
Age	0.5	0.5	1	2.92 × 10^−3^	341.08	0.05	0.01	0.03	0.08
Education	0.5	0.5	0.47	0.53	0.89	0.13	0.2	−0.04	0.63
Relationship status	0.5	0.5	0.37	0.63	0.6	−0.09	0.23	−0.68	0.3
Negative Cognitive Empathy	0.5	0.5	0.42	0.58	0.72	5.39 × 10^−3^	0.05	−0.1	0.12
Positive Cognitive Empathy	0.5	0.5	0.74	0.26	2.84	0.08	0.07	−43.1	0.22
Negative Affective Empathy	0.5	0.5	1	1.36 × 10^−6^	735,130.1	−0.26	0.05	−0.35	−0.17
Positive Affective Empathy	0.5	0.5	1	2.33 × 10^−14^	4.19 × 10^+13^	0.41	0.05	0.31	0.5

## Data Availability

The raw data supporting the conclusions of this article will be made available by the author upon reasonable request.

## Supplementary Materials

The following supporting information can be downloaded at https://www.mdpi.com/article/10.3390/ejihpe15030038/s1.
