# Supplementary material for: The Dark and Light Sides of Empathy: The Clinical Relevance of the Assessment of Cognitive and Affective Empathy Across Negative and Positive Emotions
Source: Eur J Investig Health Psychol Educ. 2025 Mar 18;15(3):38. doi: 10.3390/ejihpe15030038 (PMC11941167; doi:10.3390/ejihpe15030038)
Supplement: Supplementary file 1 [file ejihpe-15-00038-s001.zip › ejihpe-3266519-supplementary.pdf]

Supplementary Table S1. Pearson correlations between the study variables (n = 786).

Variables	1	2	3	4	5	6	7	8	9	10	11	12
1. Sex	–											
2. Age	0.07	–										
3. Education	0.04	0.40 ***	–									
4. Relationship Status	-0.12 ***	0.25 ***	0.12 **	–								
5. PES Negative Cognitive Empathy	-0.14 ***	-0.03	0.07 *	0.04	–							
6. PES Positive Cognitive Empathy	-0.08 *	0.01	0.06	0.07 *	0.82 ***	–						
7. PES Negative Affective Empathy	-0.19 ***	-0.04	0.08 *	0.04	0.29 ***	0.23 ***	–					
8. PES Positive Affective Empathy	-0.14 ***	0.02	0.04	0.06	0.32 ***	0.49 ***	0.51 ***	–				
9. PHQ-4 Anxiety	-0.15 ***	-0.24 ***	-0.09 *	-0.01	0.10 **	-0.01	0.28 ***	-0.04	–			
10. PHQ-4 Depression	-0.02	-0.23 ***	-0.10 **	-0.11 **	-0.01	-0.10 **	0.14 ***	-0.19 ***	0.69 ***	–		
11. PHQ-4 Total Score	-0.10 **	-0.26 ***	-0.10 **	-0.07	0.05	-0.06	0.22 ***	-0.13 ***	0.91 ***	0.92 ***	–	
12. WHO-5 Well-Being	0.10 **	0.17 ***	0.10 **	0.02	0.11 **	0.22 ***	-0.03	0.29 ***	-0.47 ***	-0.60 ***	-0.58 ***	–

Note. * $p < 0.05$; ** $p < 0.01$; *** $p < 0.001$. Sex was coded as follows: females = 1; males = 2. Education was coded as follows: primary school level education = 1; vocational education = 2; secondary education = 3; higher education degree = 4. Relationship status was coded as follows: single = 1; in a relationship = 2.

Supplementary Table S2. The best ten models for predicting anxiety symptoms (*n* = 786).

No.	Models	P(M)	P(M∣data)	BFM	BF10	R^2
1	Sex + Age + Negative Affective Empathy + Positive Affective Empathy	1.59×10^{-3}	0.28	242.14	1.00	0.18
2	Sex + Age + Negative Cognitive Empathy + Negative Affective Empathy + Positive Affective Empathy	1.98×10^{-3}	0.11	64.95	0.33	0.19
3	Sex + Age + Relationship status + Negative Affective Empathy + Positive Affective Empathy	1.98×10^{-3}	0.07	35.33	0.19	0.18
4	Sex + Age + Positive Cognitive Empathy + Negative Affective Empathy + Positive Affective Empathy	1.98×10^{-3}	0.06	32.77	0.18	0.18
5	Sex + Age + Education + Negative Affective Empathy + Positive Affective Empathy	1.98×10^{-3}	0.06	29.93	0.16	0.18
6	Sex + Age + Education + Relationship status + Negative Cognitive Empathy + Positive Cognitive Empathy + Negative Affective Empathy + Positive Affective Empathy	0.11	0.05	0.46	2.81×10^{-3}	0.19
7	Sex + Age + Relationship status + Negative Cognitive Empathy + Negative Affective Empathy + Positive Affective Empathy	3.97×10^{-3}	0.05	12.15	0.07	0.19
8	Sex + Age + Education + Negative Cognitive Empathy + Negative Affective Empathy + Positive Affective Empathy	3.97×10^{-3}	0.04	11.52	0.06	0.19
9	Sex + Age + Negative Cognitive Empathy + Positive Cognitive Empathy + Negative Affective Empathy + Positive Affective Empathy	3.97×10^{-3}	0.04	10.07	0.06	0.19
10	Sex + Age + Education + Relationship status + Negative Cognitive Empathy + Negative Affective Empathy + Positive Affective Empathy	0.01	0.03	2.50	0.01	0.19

Note. P(M) indicates prior model probabilities, whereas P(M ∣ data) indicates posterior probabilities of the models considered. BF_M indicates the updating factor by which the prior model odds change into the posterior model odds. BF10 indicates Bayes factor comparing a model to the null model. R^2 indicates explained variance.

Supplementary Table S3. The best ten models for predicting depression symptoms (*n* = 786).

No.	Models	P(M)	P(M∣data)	BFM	BF10	R^2
1	Age + Negative Affective Empathy + Positive Affective Empathy	1.98×10^{-3}	0.46	420.35	1.00	0.15
2	Age + Relationship status + Negative Affective Empathy + Positive Affective Empathy	1.59×10^{-3}	0.12	87.38	0.33	0.16
3	Age + Education + Negative Affective Empathy + Positive Affective Empathy	1.59×10^{-3}	0.06	39.58	0.16	0.15
4	Age + Negative Cognitive Empathy + Negative Affective Empathy + Positive Affective Empathy	1.59×10^{-3}	0.05	29.80	0.12	0.15
5	Age + Positive Cognitive Empathy + Negative Affective Empathy + Positive Affective Empathy	1.59×10^{-3}	0.04	29.37	0.12	0.15
6	Sex + Age + Negative Affective Empathy + Positive Affective Empathy	1.59×10^{-3}	0.04	28.99	0.12	0.15
7	Age + Education + Relationship status + Negative Affective Empathy + Positive Affective Empathy	1.98×10^{-3}	0.03	14.18	0.06	0.16
8	Age + Relationship status + Negative Cognitive Empathy + Negative Affective Empathy + Positive Affective Empathy	1.98×10^{-3}	0.02	11.08	0.05	0.16
9	Age + Relationship status + Positive Cognitive Empathy + Negative Affective Empathy + Positive Affective Empathy	1.98×10^{-3}	0.02	10.94	0.05	0.16
10	Sex + Age + Relationship status + Negative Affective Empathy + Positive Affective Empathy	1.98×10^{-3}	0.02	10.89	0.05	0.16

Note. P(M) indicates prior model probabilities, whereas P(M ∣ data) indicates posterior probabilities of the models considered. BF_M indicates the updating factor by which the prior model odds change into the posterior model odds. BF10 indicates Bayes factor comparing a model to the null model. R^2 indicates explained variance.

Supplementary Table S4. The best ten models for predicting well-being (*n* = 786).

No.	Models	P(M)	P(M∣data)	BFM	BF10	R^2
1	Sex + Age + Positive Cognitive Empathy + Negative Affective Empathy + Positive Affective Empathy	1.98×10^{-3}	0.19	115.92	1.00	0.17
2	Sex + Age + Education + Positive Cognitive Empathy + Negative Affective Empathy + Positive Affective Empathy	3.97×10^{-3}	0.11	31.24	0.30	0.17
3	Sex + Age + Education + Relationship status + Negative Cognitive Empathy + Positive Cognitive Empathy + Negative Affective Empathy + Positive Affective Empathy	0.11	0.11	0.99	0.01	0.17
4	Sex + Age + Education + Relationship status + Positive Cognitive Empathy + Negative Affective Empathy + Positive Affective Empathy	0.01	0.08	6.06	0.06	0.17
5	Sex + Age + Relationship status + Positive Cognitive Empathy + Negative Affective Empathy + Positive Affective Empathy	3.97×10^{-3}	0.07	18.70	0.19	0.17
6	Sex + Age + Education + Negative Cognitive Empathy + Positive Cognitive Empathy + Negative Affective Empathy + Positive Affective Empathy	0.01	0.06	4.78	0.05	0.17
7	Sex + Age + Negative Affective Empathy + Positive Affective Empathy	1.59×10^{-3}	0.06	40.90	0.41	0.16
8	Sex + Age + Negative Cognitive Empathy + Negative Affective Empathy + Positive Affective Empathy	1.98×10^{-3}	0.06	31.72	0.32	0.17
9	Sex + Age + Negative Cognitive Empathy + Positive Cognitive Empathy + Negative Affective Empathy + Positive Affective Empathy	3.97×10^{-3}	0.06	14.79	0.15	0.17
10	Sex + Age + Relationship status + Negative Cognitive Empathy + Positive Cognitive Empathy + Negative Affective Empathy + Positive Affective Empathy	0.01	0.04	2.90	0.03	0.17

Note. P(M) indicates prior model probabilities, whereas P(M ∣ data) indicates posterior probabilities of the models considered. BF_M indicates the updating factor by which the prior model odds change into the posterior model odds. BF10 indicates Bayes factor comparing a model to the null model. R^2 indicates explained variance.
